# Developmental Stage-Specific Regulation of the Circadian Clock by Temperature in Zebrafish

**DOI:** 10.1155/2014/930308

**Published:** 2014-03-27

**Authors:** Kajori Lahiri, Nadine Froehlich, Andreas Heyd, Nicholas S. Foulkes, Daniela Vallone

**Affiliations:** ^1^Institute of Toxicology and Genetics, Karlsruhe Institute of Technology, Hermann-von-Helmholtz-Platz 1, 76344 Eggenstein-Leopoldshafen, Germany; ^2^Institute of Functional Interfaces, Karlsruhe Institute of Technology, Hermann-von-Helmholtz-Platz 1, 76344 Eggenstein-Leopoldshafen, Germany; ^3^Tuebingen Hearing Research Centre, Section Physiological Acoustics and Communication, Department of Otolaryngology, Head and Neck Surgery, University of Tuebingen, 72076 Tuebingen, Germany

## Abstract

The circadian clock enables animals to adapt their physiology and behaviour in anticipation of the day-night cycle. Light and temperature represent two key environmental timing cues (zeitgebers) able to reset this mechanism and so maintain its synchronization with the environmental cycle. One key challenge is to unravel how the regulation of the clock by zeitgebers matures during early development. The zebrafish is an ideal model for studying circadian clock ontogeny since the process of development occurs *ex utero* in an optically transparent chorion and many tools are available for genetic analysis. However, the role played by temperature in regulating the clock during zebrafish development is poorly understood. Here, we have established a clock-regulated luciferase reporter transgenic zebrafish line (Tg (−3.1) *per1b*::luc) to study the effects of temperature on clock entrainment. We reveal that under complete darkness, from an early developmental stage onwards (48 to 72 hpf), exposure to temperature cycles is a prerequisite for the establishment of self-sustaining rhythms of *zfper1b*, *zfaanat2*, and *zfirbp* expression and also for circadian cell cycle rhythms. Furthermore, we show that following the 5–9 somite stage, the expression of *zfper1b* is regulated by acute temperature shifts.

## 1. Introduction

Most animals and plants adjust their physiology and behaviour according to the time of the day. These changes represent adaptations to regular environmental changes that result directly or indirectly from the rotation of our earth on its axis. These biological rhythms persist even under constant conditions and are controlled by a highly conserved endogenous clock or pacemaker [1]. The period length of the clock-generated rhythms is not precisely 24 h and so they are termed circadian (circa = around, diem = one day). Daily resetting of the clock by environmental signals such as changes in light, temperature, and nutrient availability (so called “zeitgebers”: time givers) is therefore essential to ensure synchronization with the environment [2]. At its simplest level, the clock can be considered to be composed of three parts: a central, cell autonomous pacemaker that generates the circadian rhythm; an input pathway whereby zeitgebers are perceived and adjust the phase of the pacemaker; and finally an output pathway through which the pacemaker regulates a diversity of physiological processes. The basic organization of the central clock mechanism itself appears to be highly conserved through evolution. Many clock components represent transcriptional regulators that are organized in transcription-translation feedback loops. Characteristic delays in certain steps of this mechanism confer the relatively long (24 hours) duration of one cycle [3].

Historically, most attention has been focused on how light regulates the circadian clock and the description of the light input pathway. However, temperature also has a profound effect on circadian clock function. Daily temperature cycles as well as acute shifts of temperature have been well documented to set the phase of the clock rhythm [4]. Furthermore, one of the most fundamental properties of the clock is that the period length of its rhythm remains relatively constant over a range of temperatures, so-called “temperature compensation” [4, 5]. Outside the range of temperature compensation, the clock characteristically arrests at a certain phase [6, 7]. The physiological temperature range for rhythmicity lies well within the temperature range permissive for growth. The molecular basis of these effects of temperature has come primarily from studies of ectothermic organisms including* Neurospora*, lizards, and* Drosophila* [8–11]. Fish have proved to be ideally suited to study the specific effects of temperature cycles on the circadian clock [12, 13]. Although in endothermic organisms temperature changes have little influence on the central pacemaker, fluctuations in body temperature appear to synchronize and thereby sustain circadian rhythms in peripheral tissues [14–16].

The zebrafish naturally inhabits shallow water habitats where it is likely to experience daily changes in water temperature. Using this species as a model, we have previously explored the molecular basis of temperature compensation as well as entrainment of the circadian clock by small temperature shifts [17]. We revealed that the amplitude of clock gene cycling expression in zebrafish is tightly dependent on the ambient temperature. Furthermore, small temperature shifts result in acute changes in the mRNA expression of a subset of clock genes. Thus, in the case of zebrafish peripheral tissues, changes in clock gene transcription appear to contribute to the response of the clock to temperature changes.

When during development does the circadian clock start to function? When is rhythmic circadian clock gene expression first detected? The zebrafish is an ideal model system to understand circadian clock ontogeny since the process of development occurs* ex utero* in an optically transparent chorion and many tools for functional analysis are available. In a study using a zebrafish* per3*-luciferase transgenic line, bioluminescence rhythms were first detected in larvae at 4-5 days postfertilization (dpf) [18]. Other studies documenting the appearance of key clock outputs such as circadian rhythms of S-phase and pineal production of melatonin have revealed the emergence of rhythms from 24 to 36 hours postfertilization (hpf) [19–22]. In all cases, the presence of an LD cycle or a single transition between dark and light from 24 hpf is a prerequisite for the emergence of clock rhythmicity. A detailed study of rhythmic clock gene expression in developing embryos revealed that clock gene expression rhythms are detected very early during development at the single cell level; however, these cell clocks are asynchronous. The effect of exposure to LD cycles is to synchronize the single cell clocks [23].

The role played by temperature in clock entrainment during early zebrafish development is relatively poorly understood. We have established a clock-regulated luciferase reporter transgenic line (Tg (−3.1)* per1b*::luc) and then used this as a model to explore the origins of temperature entrainment in detail.

## 2. Materials and Methods

### 2.1. Fish Care, Treatment, and Ethical Statements

The zebrafish Tuebingen strain was raised and bred according to standard procedures [24] in a recirculating water system under 14 hours light and 10 hours dark cycles at 28°C and fed twice per day.

All zebrafish husbandry and experimental procedures were performed in accordance with the German animal protection standards (Animal Protection Law, BGBl. I, 1934 (2010)) and were approved by Local Animal-Protection Committee, Regierungspräsidium Karlsruhe, Germany (License number BrdU treatments Az.:35-9185.81/G-130/12 and general license for fish maintenance and breeding Az.:35-9185.64).

### 2.2. Establishment of the Tg (−3.1)* per1b*::luc Transgenic Line

Microinjections for the establishment of the transgenic line were made at the one cell stage as described [24]. Embryos were injected with the pCMV-I-Sce I expression vector encoding a modified I-Sce I meganuclease (276 amino acids) [25] together with the pBluescript* Per1b-luc*-I-SceI plasmid (containing a 3.1kb fragment of the* zfper1b (per4)* promoter [26] cloned upstream of a luciferase reporter). The latter plasmid contains two I-SceI recognition sites for the meganuclease. Embryos were screened for bioluminescence by raising them from 1 to 6 dpf under LD (12 h : 12 h) cycles and then transferring them to 96-multiwell plates in the presence of 0.5 mM beetle luciferin potassium salt solution (Promega) as described elsewhere [27]. Counts were measured on a Perkin Elmer luminescence counter (VICTOR Light 1420) during the first half of the day when* zfper1b* expression was predicted to be at its highest [26]. Positive germline transmitting founders were outcrossed and heterozygous animals were raised.

### 2.3. Temperature and Lighting Conditions

All the experiments were performed under controlled lighting and temperature conditions. The precise conditions for each experiment are indicated either in the corresponding results or figure legends sections. For temperature cycle experiments, larvae maintained in 96-well plates or in 25 cm^2^ flasks were submerged in a 60-liter water bath with circulating heating and cooling units (Lauda, Lauda-Königshofen, Germany) maintained in complete darkness. Temperature cycles were generated by controlling the heating and cooling units using Wintherm plus software (Lauda). During the cold/warm phase transition periods, temperature changes of 1°C were set to occur over 15 minutes. For light/dark cycle experiments, larvae in 96-well plates or in flasks were submerged in a 60-liter thermostat-controlled water bath illuminated with a tungsten light source (20 *μ*W/cm^2^).

### 2.4. Real-Time Bioluminescence Assay

For all experiments, single embryos were aliquoted into individual wells of a 96-multiwell plate (Nunc) in E3 media (without Methylene Blue) supplemented with 0.5 mM beetle luciferin, potassium salt solution (Promega) and the plate was then sealed using an adhesive “Top Seal” sealing sheet (Packard). Plates were then subjected to different temperature or lighting conditions and bioluminescence from whole embryos and larvae was assayed using a Packard Top-count NXT scintillation counter (2-detector model, Packard) or an EnVision multilabel counter (Perkin Elmer). Bioluminescence data was analyzed using the Import and Analysis Macro (I&A, Plautz and Kay, Scripps) for Microsoft Excel or CHRONO software [28].

### 2.5. RNA Extraction, RNAse Protection Assays (RPA), and Quantitative Real-Time PCR (qRT-PCR)

Total RNA samples were extracted from whole larvae using Trizol RNA isolation reagent (GIBCO-BRL) according to the manufacturer's instructions. RNAse protection assays (RPA) were performed as previously described [26]. The riboprobes for* zfper1b (per4), zfirbp*, and the loading control,* zf*β*-actin*, have been described previously [26, 29]. Autoradiographic images were quantified with the aid of Scion Image (NIH) software. The relative expression levels (percentage) are plotted on the *y*-axis. *β*-*actin* levels were used to standardize the results. The highest band intensity in each experiment was arbitrarily defined as 100% and then all other values were expressed as a percentage of this value. All experiments were performed in triplicate and error bars denote the standard deviation.

Quantitative Real-Time RT-PCR (qRTPCR) analysis was performed using a StepOnePlus Real-Time RT-PCR System (Applied Biosystems) and SYBR Green I fluorescent dye (Qiagen). Relative expression levels were normalized using **β*-actin*. The relative levels of each mRNA were calculated using the 2^−ΔΔCT^ method. For* zfper1b* the primer sequences used were F: CCGTCAGTTTCGCTTTTCTC and R: ATGTGCAGGCTGTAGATCCC, while for* zf*β*-actin* the primer sequences were F: GCCTGACGGACAGGTCAT and R: ACCGCAAGATTCCATACCC.

### 2.6. BrdU Incorporation Assay

Larvae were incubated for 15 minutes in 10 mM BrdU fish water solution at different time points during a temperature or light/dark cycle and fixed in 4% PAF in PBS.

The anti-BrdU (1 : 200 Acris BM6048) and the alkaline phosphatase anti-mouse (1 : 500 Vector AP 2000) antibodies were used as first and secondary antibodies, respectively. Staining and data analysis for BrdU incorporation in the skin were carried out as described previously [30].

### 2.7. Whole-Mount* In Situ* Hybridization

Larvae previously exposed to temperature or light cycles were fixed in 4% paraformaldehyde in PBS at different time points during 24 hours.* In situ* hybridization was carried out as previously described [31] using the* zfaanat2* probe [20] that was labeled with digoxigenin RNA-labeling mix (Roche).

### 2.8. Statistical Analysis

Data were analyzed by one-way or two-way analysis of variance (ANOVA) followed by Bonferroni's multiple comparison tests using the GraphPad Prism 4.0 for Windows (Graph Pad Software, http://www.graphpad.com/). Two-way ANOVA were performed using SPSS v 16.0 for Windows (IBM, USA). Cosinor analyses were performed using COSINOR v3.0.2 software (Professor Antoni Diez-Noguera, University of Barcelona). All the results were expressed as mean +/− SEM. *P* < 0.05 was considered statistically significant.

## 3. Results

### 3.1. Establishment and Characterization of the Zebrafish Tg (−3.1)* per1b*::luc Transgenic Line

**Figure 1:**
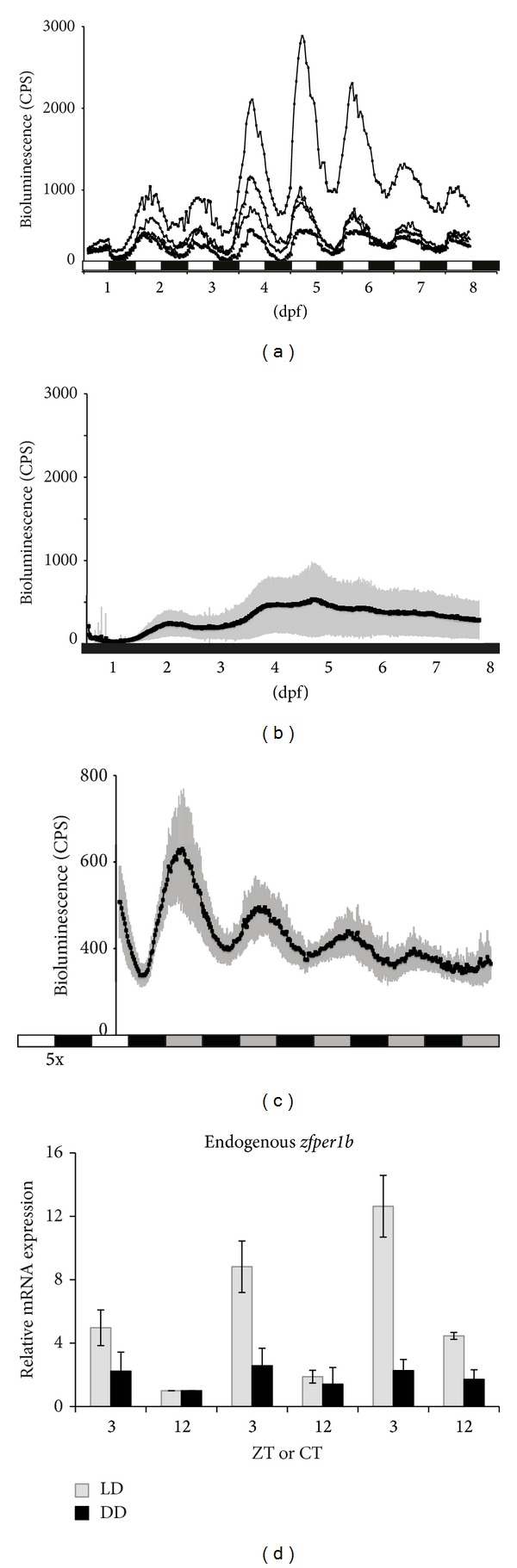
Characterization of the zebrafish Tg (−3.1)* per1b*::luc transgenic line. (a) Bioluminescence in single Tg (−3.1)* per1b*::luc transgenic embryos during development from 3 hrs postfertilization and under light/dark cycles (12 : 12 at 25°C). Traces of 4 representative animals are shown. Below, white and black horizontal bars indicate the duration of the 12 hours light (white) and 12 hours dark (black) periods of the LD cycles. (b) Mean values of bioluminescence expression profiles from transgenic larvae entrained for 5 days in LD cycles at 25°C and then monitored in constant darkness (DD) and constant temperature (25°C). Below, white, grey, and black horizontal bars represent the 12 hours light, subjective day and dark (or subjective night) periods, respectively. Before transfer to constant darkness, the embryos were previously exposed to 5 LD cycles (5×). (c) Mean of bioluminescence expression profiles from transgenic larvae raised for 8 days in DD conditions (black horizontal bar). (d) Peak (ZT3) and trough (ZT12) values of endogenous rhythmic* zfper1b* expression in embryos raised under DD (black vertical bars) or LD (grey vertical bars) cycles from 2 to 4 dpf. The expression levels are represented as fold induction values with respect to the lowest value for each set of samples (ZT12 of the second day for both sets). Each time point represents a pool with a minimum of *n* = 20 larvae.

Zebrafish embryos were injected with the* pBPer1b-luc-I-SceI* construct containing a 3.1kb fragment of the* zfper1b* promoter cloned upstream of a firefly luciferase reporter gene flanked by two I-SceI recognition sites (Figure S1(A)) (see Supplementary Material available online at http://dx.doi.org/10.1155/2014/930308). This construct was coinjected with a plasmid expressing the I-SceI meganuclease enzyme (Figure S1(B)). Out of 280 luciferase positive embryos screened for germline transmission, 3 bioluminescence-positive offspring were obtained. From these we established one line: Tg (−3.1)* per1b*::luc. We firstly analyzed the profile of bioluminescence expression of entire transgenic embryos exposed to light/dark cycles (LD: 12 hours light/12 hours dark) during the first week of development (Figure 1). A significant daily rhythm of bioluminescence was already visible from 2 dpf (days-post-fertilization) (Figure 1(a)). A one-way ANOVA (*P* < 0.0001) followed by a Cosinor analysis of the mean profile of the Tg (−3.1)* per1b*::luc embryos showed the presence of significant periodic oscillations (*P* < 0.00001), although during the first two days of development some variability between individual embryos was observed in the rhythm amplitude and in the precise timing of peaks of expression (22.8% < robustness > 54.9%; 23.8 < period > 25.1 and ZT 2.34 < peak < ZT 3.49). As predicted for expression of the core clock element* zfper1b*, the light entrained bioluminescence rhythm of the Tg (−3.1)* per1b*::luc larvae persisted after transfer to constant darkness (DD) (Figure 1(b)) showing that changes in luciferase reporter expression were not simply driven by light (one-way ANOVA *P* < 0.0001 to test for the time effect followed by Cosinor analysis *P* < 0.000001 to test for periodic oscillation). Furthermore, consistent with previous reports, raising the transgenic larvae in the complete absence of light (DD) resulted in no significant bioluminescence oscillation (Figure 1(c) and *P* > 0.05 for Cosinor analysis), although a significant time effect was observed at 2 and 5 dpf (one-way ANOVA *P* < 0.001 for the time effect). Changes in the basal level of expression of bioluminescence observed at 2 dpf and between 4 and 5 dpf most likely reflect changes in basal expression of* zfper1b* associated with embryonic development.

To validate our transgenic line, we verified that the bioluminescence rhythm of the Tg (−3.1)* per1b*::luc line measured under LD cycles matched the endogenous mRNA expression pattern of the* zfper1b* gene assayed in whole larval RNA extracts (Figure 1(d)). We observed a significant daily oscillation of endogenous* zfper1b* expression from 2 dpf with peak points around ZT3 and trough points around ZT12 (one-way ANOVA *P* < 0.001 for the time effect, *P* < 0.0001 followed by Cosinor analysis, and *P* < 0.000001 for periodic oscillation). Thus, we have established an* in vivo* model that accurately reports endogenous clock gene expression and thereby can facilitate the study of clock function during early development.

### 3.2. Clock Entrainment by Temperature Cycles in Developing Zebrafish Embryos

**Figure 2:**
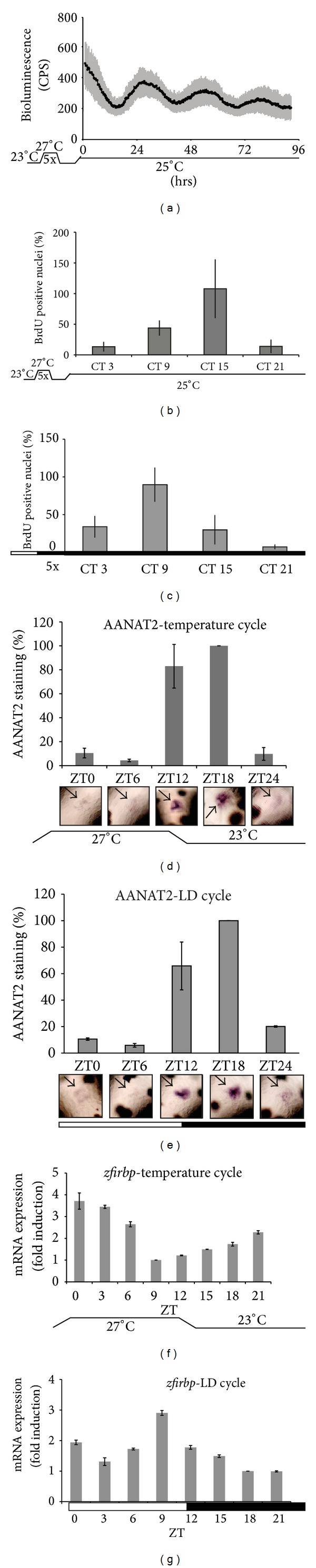
Clock entrainment by temperature cycles in developing zebrafish embryos. (a) Mean values of bioluminescence expression profiles from transgenic larvae entrained for 5 days under 4°C temperature cycles (23–27°C) in constant darkness and then monitored at a constant temperature of 25°C. ((b)-(c)) Whole-mount staining for BrdU incorporation in skin cells of larvae previously entrained by 5× temperature (b) and 5× LD (c) cycles and then sampled during the first day after transfer to constant conditions (darkness and 25°C). The number of BrdU positive nuclei (S-phase nuclei), normalized as percentage (%) of the highest experimental value, is plotted on the *y*-axis. ((d)-(e)) Rhythmic expression of the circadian clock-regulated* zfaanat2* gene in 5 dpf zebrafish larvae under 4°C temperature (d) or light/dark (e) cycles. In each panel, representative* zfaanat2 in situ* hybridization images and quantification graphs from independent experiments (each time point included a minimum of 20 larvae) are shown. On the *y*-axis, expression levels are expressed as % of the highest level measured in each set. Black arrows indicate the position of the* zfaanat2* mRNA signal in the pineal gland. ((f)-(g)) Quantification of the RNAse protection analysis of* zfirbp* expression in whole larva mRNA extracts in larvae entrained under 5× temperature (f) or 5× LD (g) cycles. In each panel, the *x*-axes indicate the times of measurement (hrs), the circadian (CT), or the zeitgeber (ZT) times. On the *y*-axis, expression levels are expressed as fold induction compared to the lowest level measured in the set. In each panel, points are plotted as the means of three independent experiments ± SEM. The black horizontal lines beneath panels (a) and (b) indicate constant temperature while the temperature cycles in panels (a), (b), (d), and (f) are indicated. Black and white bars beneath panels (c), (e), and (g) indicate the dark and light periods of the lighting regimes, respectively.

Many previous reports have documented that temperature changes can serve as an efficient zeitgeber for the fish circadian clock [12, 13, 17]. Can temperature cycles entrain the clock in developing zebrafish embryos? To address this question, we raised Tg (−3.1)* per1b*::luc embryos under 4°C temperature cycles (23°C–27°C) in constant darkness from 1 dpf for 5 days and then we monitored bioluminescence at a constant 25°C (Figure 2(a)). We observed robust circadian rhythms of bioluminescence which dampened progressively during the assay period (5−9 dpf) (*P* < 0.000001 for periodic oscillation with free running period tau = 25.1; one-way ANOVA *P* < 0.001 for the time effect).

### 3.3. Clock-Regulated Outputs Entrained by Temperature Cycles during Zebrafish Development

The circadian clock directs a wide variety of output pathways. We have previously implicated both the circadian clock and light in the control of cell cycle timing in zebrafish [30, 32]. We wished to test whether temperature cycles, in the absence of light, would also entrain cell cycle rhythms in zebrafish embryos. Temperature itself affects the rate of cell division and other biochemical processes. In order to avoid the complicating effect of temperature driven changes in cell cycle progression we tested the timing of cell cycle in free running conditions at a constant temperature following a period of temperature cycle entrainment. Therefore, we entrained zebrafish larvae by exposure to a 4°C and 24 hours temperature cycle (27°C–23°C) from 1 to 5 dpf and then shifted them to a constant temperature of 25°C for assay. In parallel, as a control, larvae were raised until 5 dpf under LD cycles and then shifted to DD conditions. Both sets of 6 dpf larvae were BrdU labelled for 15 minutes at different circadian time points (CT 3, 9, 15, and 21) and then stained for the presence of BrdU positive (S-phase) nuclei. Positive skin cell nuclei were counted between the posterior tip of the swim bladder and the anus as previously described [30]. Both sets of larvae showed a circadian rhythm in the number of S phase-positive nuclei (Figures 2(b) and 2(c)) with a peak around CT 15 or CT 9 for larvae under temperature or light cycles, respectively (Figures 2(b) and 2(c)) (one-way ANOVA *P* < 0.0001 for the time effect followed by *P* < 0.0001 for periodic oscillation for both temperature and light zeitgebers). Thus, entrainment of the clock by temperature cycles as well as LD cycles results in circadian cell cycle rhythms in developing larvae.

We next examined whether entrainment by temperature cycles could also establish a clock-regulated rhythm of melatonin synthesis. We examined the expression of* zfaanat2* mRNA in the pineal gland, which encodes the rate-limiting enzyme in melatonin biosynthesis [33, 34]. Embryos were raised from 1 to 5 dpf in 4°C temperature cycles in the absence of light and control siblings were raised under LD cycles at a constant temperature. The expression of* zfaanat2* mRNA was detected in the pineal gland of both sets of larvae. In the temperature cycle larvae (Figure 2(d)),* zfaanat2* expression cycles in a circadian fashion with high expression levels during the transition from warm to cold (ZT12 and ZT18) and low levels during the transition from cold to warm temperatures (ZT0/24 and ZT6) (one-way ANOVA *P* < 0.001 for the time effect followed by *P* < 0.0001 for periodic oscillation). In the LD set, high expression levels are observed during the day-night transition (ZT 12 and ZT 18) and low to undetectable levels during the night-day transition (ZT 0/24 and ZT 6), consistent with previous reports [20] (Figure 2(e)) (one-way ANOVA *P* < 0.001 for the time effect followed by *P* < 0.0001 for periodic oscillation). Thus, in the absence of light, rhythmic* zfaanat2* expression can be established by exposure to a temperature cycle.

Finally, we examined the expression of interphotoreceptor retinoid-binding protein (*zfirbp*) mRNA in whole body RNA extracts prepared from our larvae raised under 4°C temperature cycles compared with LD cycles (Figures 2(f) and 2(g)). Consistent with previous reports documenting* zfirbp* expression in the retina and pineal gland [23], in LD larvae (Figure 2(g)) we revealed a rhythmic expression profile with a peak of expression during the light phase (peak ZT 9) (one-way ANOVA *P* < 0.0001 for the time effect followed by *P* < 0.0001 for periodic oscillation). In larvae entrained by temperature cycles, we also observed rhythmic expression of* zfirbp.* The highest expression was detected at the transition between the cold and warm phases (Figure 2(f)) (one-way ANOVA *P* < 0.0001 for the time effect followed by *P* < 0.0001 for periodic oscillation). Thus, in photoreceptive structures, temperature cycles can substitute for LD cycles to entrain rhythmic expression of clock output genes.

### 3.4. Clock Entrainment by Temperature Cycles Is Dependent on the Developmental Stage

**Figure 3:**
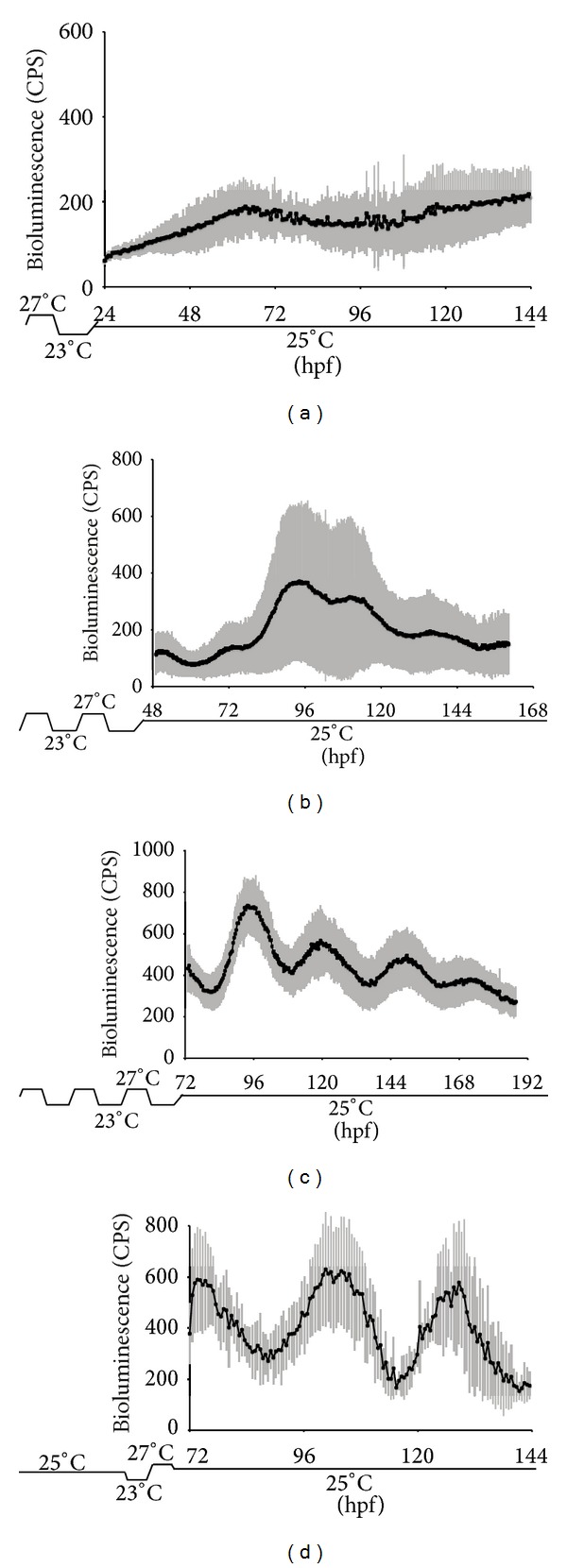
Clock entrainment by temperature cycles is dependent on the developmental stage. ((a)–(c)) Mean values of bioluminescence profiles of transgenic embryos raised under 4°C temperature cycles (23–27°C) for (a) 1 day (0–24 hpf), (b) 2 days (24–48 hpf), or (c) 3 days (48–72 hpf) and then monitored for 5 days at a constant 25°C. (d) Mean values of bioluminescence profiles of transgenic embryos raised at a constant 25°C for the first two days of development (0–48 hpf) and then subjected to a single 4°C temperature cycle (23–27°C) during the third day (48–72 hpf) before monitoring for three days at 25°C. In each panel, standard deviation of bioluminescence measurements between animals is plotted as grey vertical lines above each point. Each panel shows the mean values of 96 larvae (1 × 96 multiwell plate).

When during development are temperature cycles able to entrain the circadian clock? To tackle this question, we used our clock reporter transgenic line. Tg (−3.1)* per1b*::luc embryos from 1 hour postfertilization (1hpf) were subjected to 1, 2, or 3 temperature cycles (23°C–27°C) in the absence of light. Then the larvae were tested in a bioluminescence assay at a constant temperature. Pools of transgenic larvae raised either under 1 or 2 temperature cycles showed a mean bioluminescence profile closely resembling that observed for larvae raised under constant temperature and darkness conditions (Figures 3(a) and 3(b) and compare with Figure 1(c)) with peaks of expression observed on the 2nd and on the 4th-5th day but with a complete lack of* circa* 24 hours periodicity. Interestingly, a small subset (*circa* 10%) of individual larvae under these conditions did exhibit cycling reporter expression although with different phases and amplitudes. In contrast, in the majority of embryos exposed to 3 temperature cycles, a robust circadian rhythm of bioluminescence was established (Figure 3(c)). We next questioned whether it is the number of temperature cycles experienced that synchronizes the clock* in vivo* or whether only at a particular developmental stage are the larvae responsive to temperature signals. Thus, to address this question we raised embryos under constant temperature and constant darkness and then exposed them to a single temperature cycle at 3 dpf before immediately assaying bioluminescence under constant conditions (Figure 3(d)). Interestingly, one temperature cycle delivered at 3 dpf results in circadian cycling comparable to that induced by exposure to 3 consecutive temperature cycles. This result indicates that the developmental stage is an important factor in defining the sensitivity of the circadian clock to entrainment by temperature cycles.

### 3.5. An Acute Temperature Shift Regulates Clock Gene Expression during Early Development from the 5–9 Somites Stage

**Figure 4:**
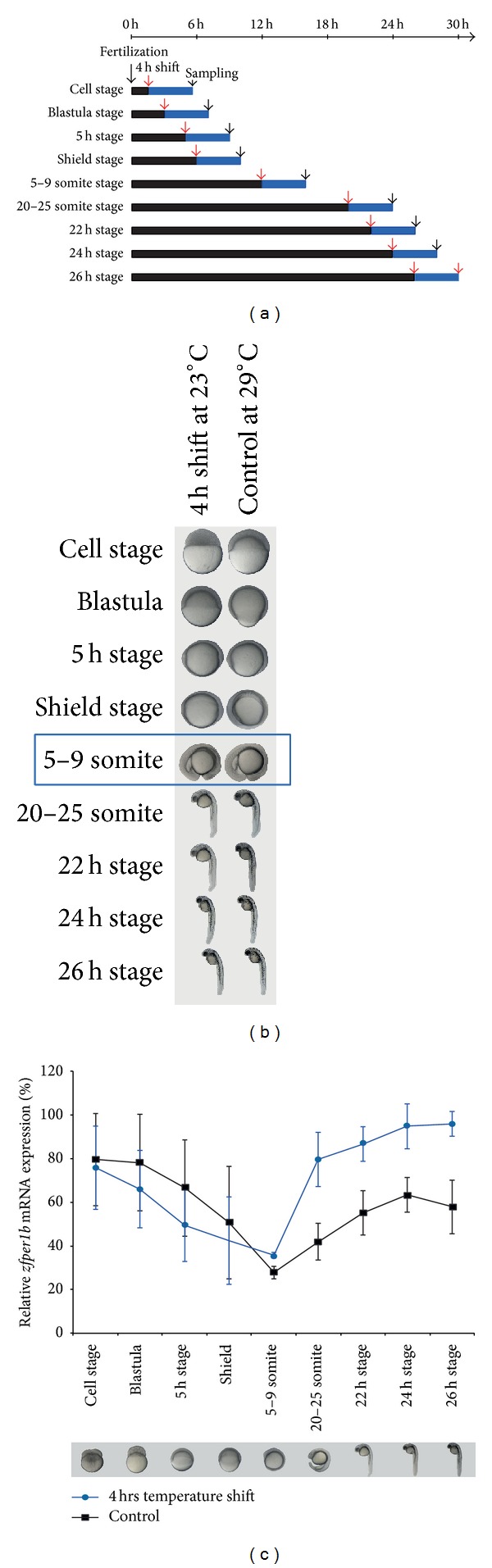
Acute temperature shift regulates* zfper1b* expression during early development. (a) Schematic representation of the experimental design. After fertilization, embryos were raised under a constant 29°C and at different developmental stages were acutely shifted for 4 h to 23.5°C (red arrows) (circa 20 embryos for each experimental group). Then mRNA from temperature shifted groups and a control group maintained at a constant 29°C were isolated (black arrows). (b) Images of embryos raised at 29°C (control) or 4 hrs after a temperature shift applied at different developmental stages. The developmental stage where the temperature shift induced* zfper1b* expression is indicated by a blue box. (c) Expression level of* zfper1b* measured in the different groups of embryos by qRT-PCR (control in black and temperature shift groups in blue).

Previous studies have implicated differential maturation of elements of the circadian clock during early embryonic development. Is there evidence of temperature-dependent clock gene expression prior to the entrainment of the clock by temperature? We have previously reported a rapid response of* zfper1b* transcription to acute temperature shifts in the Pac-2 cell line. A rapid temperature decrease results in sustained induction, while a temperature increase leads to a significant downregulation of* zfper1b* expression [17]. We wished to test at which stage of development this temperature-induced change in* zfper1b* expression is first detected. We raised embryos immediately after fertilization in constant darkness at 29°C until different defined stages during early development. We then subjected them to a single temperature shift to 23.5°C for 4 hours before immediately sacrificing them for RNA extraction and assay of* zfper1b* mRNA levels (Figure 4). The* zfper1b* transcript is clearly maternally inherited, being detected before the onset of zygotic transcription. Subsequently, during the initial stages of development starting from the early cell stage to shield stage, the expression levels of* zfper1b* slowly decreased and did not show any significant response to the temperature shift (Figure 4(c)). Starting from the 5–9 somites stage the expression levels of* zfper1b* started to increase significantly (blue trace and one-way ANOVA *P* < 0.001) following the temperature shift when compared to control embryos maintained for the entire experiment at 29°C (black trace). These results indicate that following the 5–9 somites stage of development, the expression of key core clock elements becomes responsive to temperature changes.

## 4. Discussion

The zebrafish represents a valuable vertebrate model for studying the origins and regulation of circadian clock function during embryonic development. Such studies are technically more difficult to perform and interpret in placental mammals such as the mouse. Furthermore, the use of bioluminescent clock reporters has been extensively employed for noninvasive analysis of the clock in a broad range of model species. The use of such an* in vivo* luciferase assay to monitor gene expression has allowed us to measure circadian clock gene expression in individual embryos and larvae. One of the surprising results has been the considerable interindividual variability in rhythm amplitude, basal expression levels, and also the general expression profile. For these reasons, we have expressed results as the mean of individual traces from between 62 and 79 individuals. This variability from embryo to embryo might reflect real differences at the level of gene expression regulation and signal transduction—which are not reflected by differences at the morphological level. It is tempting to speculate that such differences, if maintained during development, might ultimately confer different circadian clock properties on individual fish.

Our results have revealed several properties of the developing circadian clock mechanism. Firstly, in the absence of entraining signals such as light or temperature cycles, most larvae show a normal morphological development and fail to establish overt circadian rhythms. Thus our results agree with previous studies addressing this issue [18, 20–23] and disagree with one early report claiming maternal inheritance of the circadian clock [35]. However, our results have shown that* zfper1b* gene expression is not constant during the development of larvae under constant conditions. The* zfper1b* expression level gradually increases from fertilization until a first peak at 2 dpf and then subsequently a second peak at circa 4-5 dpf (25°C) corresponding to the hatching time. It is tempting to speculate that these changes in expression reflect key developmental events.

Exposure to temperature cycles during the first 2 days of development fails to establish a circadian rhythm. This contrasts with the situation in zebrafish Pac-2 cells, where a single temperature cycle is sufficient to establish robust rhythms of clock gene expression [17]. One could speculate that the lack of response of the embryonic clock to 2 cycles of entrainment might reflect incompatibility with the high levels of cell proliferation or low levels of cell differentiation that characterizes this developmental period. The finding that the phase of the circadian clock is faithfully inherited when daughter cells divide and that cell cycle progression does not appear to interfere with the clock [36] would tend to argue against high levels of cell proliferation being responsible for the lack of entrainment during the first 2 days. However these reports have been made with cultured differentiated cells. It therefore remains to be tested whether the same applies for embryonic cells* in vivo*. Alternatively, expression of certain clock components or key elements of temperature sensing signal transduction pathways may not yet have matured prior to the second day of development. When larvae are raised under constant conditions, the third day of development immediately follows the first peak of period gene expression, again suggesting that this may reflect a landmark in the establishment of the clock at the whole animal level. Interestingly, a single temperature shift delivered during the first 12 hours of development was previously reported to elicit circadian rhythms of endogenous* zfper1* mRNA expression [23]. Differences between this report and our findings may result from the fact that our transgenic assay specifically reports changes in transcription while assays of endogenous mRNA levels may also reflect regulated mRNA stability. Thus, potentially temperature shifts may regulate gene expression at multiple levels.

Interestingly, previous studies have reported that single light/dark transitions as early as 24 hpf are sufficient to initiate circadian clock rhythmicity in the pineal gland [22]. However, our results that are based on clock gene expression at the whole animal level suggest a much later maturation of temperature cycle entrainment. This may reflect a real difference in the developmental appearance of clock entrainment by these two zeitgebers. Alternatively, there may be tissue specific differences in the maturation of circadian clock entrainment mechanisms. Our results differ from the previously published data for the* per3*-luc transgenic line [18], where fish exhibited circadian rhythms of bioluminescence only when subjected to more than 4xLD cycles. This may indicate that expression of the* zfper3* and* zfper1b* genes differs during development. In this regard, there is already evidence that the spatiotemporal expression pattern of* zfper2* and* zfper3* differs [37], suggesting differential roles for clock gene family members during the development of various tissues. This may point to cell type-specific roles for the clock or alternatively noncircadian clock functions for certain clock genes.

Our laboratory has previously demonstrated that the circadian clock regulates the timing of S-phase of the cell cycle [30, 32]. Here we have demonstrated that S-phase rhythms are also observed in larvae that have been exposed to a temperature cycle and then transferred to constant temperature. An apparent phase difference between the LD and temperature cycle-entrained cell cycle rhythms may reflect differences in the timing of how light/dark and high-low temperature transitions affect the core clock and, in turn, the cell cycle control machinery. Temperature cycle entrainment of cell cycle rhythms reinforces the idea that these are not purely light-driven but represent a circadian clock output. The cell cycle in most vertebrate species is not a temperature compensated process and so the temperature itself can influence its rate of progress. Given that the clock represents a temperature compensated mechanism, the coupling of temperatures cycles, clock entrainment, and the timing of the cell cycle may represent a mechanism to constrain the rate of cell proliferation under different environmental temperature conditions and thereby control the rate of development. Indeed, recent studies have revealed a strong impact of exposure to temperature as well as light cycles on the timing of hatching as well as the subsequent larval performance in zebrafish [38, 39].

Our results have revealed that circadian rhythms of* zfaanat2* expression, in the photoreceptive pineal gland, can also mature during development in the complete absence of light, upon exposure to temperature cycles. We have also revealed that temperature cycles result in the establishment of rhythmic* zfirbp* expression in larvae. The function of IRBP seems to be linked with the visual cycle trafficking of 11-*cis* retinal and all-*trans* retinol between the photoreceptors and the retinal epithelium. The* irbp* gene is expressed early during rodent retinal development and is upregulated before the expression of opsins [40]. The temporal and spatial expression patterns of the* zfirbp* gene in zebrafish are consistent with a role in retinal development and suggest coordination of retinal pigment epithelium and photoreceptor differentiation [41]. There has been so far no evidence that temperature can play a role in the regulation of expression of this gene.

## 5. Conclusions

Together, our results document a developmental maturation of the clock's response to temperature and set the stage for future work aimed at exploring how much the mechanisms of entrainment via temperature cycles and LD cycles are comparable.

## Supplementary Material

Schematic representation of the two plasmids co-injected at the one cell stage in zebrafish embryos to establish the zebrafish Tg (−3.1) *per1b*::luc transgenic line.
