# Supplementary figures and images for: Developmental Stage-Specific Regulation of the Circadian Clock by Temperature in Zebrafish
Source: Biomed Res Int. 2014 Mar 27;2014:930308. doi: 10.1155/2014/930308 (PMC3984786; doi:10.1155/2014/930308)

## A pBPer1b-luc-I-SceI

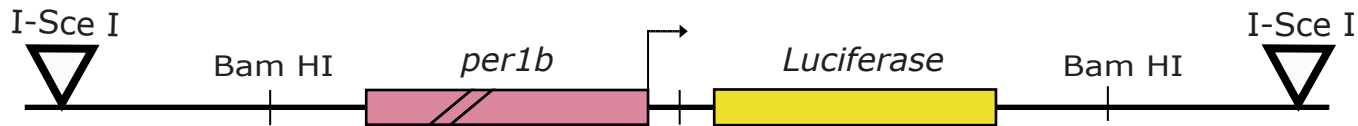

## B pBluescript SK- CMV-I-Sce I meganuclease enzyme

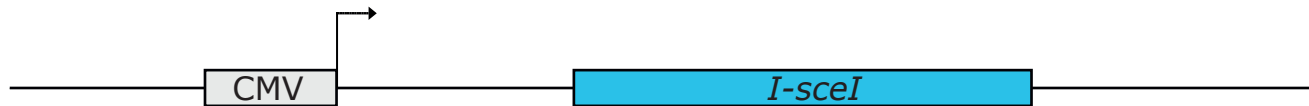

Supplement: Supplementary file 1 — Schematic representation of the two plasmids co-injected at the one cell stage in zebrafish embryos to establish the zebrafish Tg (−3.1) per1b::luc transgenic line. [file 930308.f1.pdf]
